# Optimal Environmental Conditions and Anomalous Ecosystem Responses: Constraining Bottom-up Controls of Phytoplankton Biomass in the California Current System

**DOI:** 10.1038/srep27612

**Published:** 2016-06-09

**Authors:** Michael G. Jacox, Elliott L. Hazen, Steven J. Bograd

**Affiliations:** 1 Institute of Marine Sciences, University of California, Santa Cruz, CA, USA; 2 Environmental Research Division, Southwest Fisheries Science Center, NOAA, Monterey, CA, USA

## Abstract

In Eastern Boundary Current systems, wind-driven upwelling drives nutrient-rich water to the ocean surface, making these regions among the most productive on Earth. Regulation of productivity by changing wind and/or nutrient conditions can dramatically impact ecosystem functioning, though the mechanisms are not well understood beyond broad-scale relationships. Here, we explore bottom-up controls during the California Current System (CCS) upwelling season by quantifying the dependence of phytoplankton biomass (as indicated by satellite chlorophyll estimates) on two key environmental parameters: subsurface nitrate concentration and surface wind stress. In general, moderate winds and high nitrate concentrations yield maximal biomass near shore, while offshore biomass is positively correlated with subsurface nitrate concentration. However, due to nonlinear interactions between the influences of wind and nitrate, bottom-up control of phytoplankton cannot be described by either one alone, nor by a combined metric such as nitrate flux. We quantify optimal environmental conditions for phytoplankton, defined as the wind/nitrate space that maximizes chlorophyll concentration, and present a framework for evaluating ecosystem change relative to environmental drivers. The utility of this framework is demonstrated by (i) elucidating anomalous CCS responses in 1998–1999, 2002, and 2005, and (ii) providing a basis for assessing potential biological impacts of projected climate change.

Eastern Boundary Current ecosystems are highly productive regimes that support rich and diverse biological communities from phytoplankton to top predators^1^,^2^. Upwelling-driven nitrate flux to the euphotic zone, forced by equatorward alongshore wind, is the foundation for the high biological productivity of these regions^3^, and changes in the upwelled nitrate supply have been invoked to explain ecosystem change on seasonal^4^ to multi-decadal^5^,^6^ timescales. Such explanations for ecosystem change typically invoke a chain of events whereby increased (decreased) upwelling leads to greater (lower) nitrate supply and subsequently enhanced (reduced) primary productivity, a paradigm that is supported by broad-scale (seasonal, regional) patterns. For example, the annual onset of persistent equatorward wind off California (i.e., the ‘spring transition’) supplies nitrate to the sunlit surface layer that in turn stimulates substantial new production^7^.

However, a growing body of literature suggests the existence of a non-monotonic relationship between alongshore wind and the biological response, in which strong winds limit productivity and phytoplankton biomass through various physical mechanisms. Huntsman and Barber^8^ describe the potential for light limitation due to deepening of the surface mixed layer at high wind speeds, and a number of modeling and observational studies cite subduction and/or offshore advection as common mechanisms for removal of nutrients and organic matter from the nearshore euphotic zone during upwelling-favorable conditions^9^,^10^,^11^,^12^,^13^,^14^,^15^,^16^. Previous studies have found that moderate wind speeds are optimal for nearshore phytoplankton populations^17^,^18^, however they are either idealized or geographically limited, and do not explicitly consider variability in the subsurface nitrate field relative to wind forcing or the interaction between the nearshore and offshore environments. Furthermore, the supply of nitrate to the surface mixed layer during upwelling can be altered not only by variability in local winds, but also by changes in the water column structure. Remote influences, especially related to basin-scale climate variability (e.g. El Niño-Southern Oscillation, Pacific Decadal Oscillation), can enhance or reduce upwelled nitrate through modification of the nitracline depth as well as the water column stratification and resultant source depth of upwelling^19^,^20^.

In this study, we use a regional ocean model to derive estimates of subsurface nitrate (based on observed temperature-salinity-nitrate relationships) and surface wind stress, and combine them with satellite chlorophyll measurements to explore physical and chemical controls on phytoplankton biomass during the California Current System (CCS) upwelling season (see Methods for details). Using data from 1998 to 2010, we define the individual and combined influences of nearshore wind stress and subsurface nitrate concentration on chlorophyll concentrations in both the nearshore and offshore environments, and use this framework to elucidate the bottom-up forcing behind three periods of highly anomalous ecosystem responses in the CCS: the delayed upwelling season of 2005, strong subarctic influence in 2002, and the El Niño/La Niña conditions of 1998–1999. We note at the outset that wind and nitrate are just two contributors to phytoplankton dynamics in the CCS, and many more (e.g., iron, ammonia, zooplankton grazing, others listed in Methods) are not considered here. However, as wind and nitrate are commonly invoked to explain bottom-up ecosystem control, we focus our analysis on them.

## Results

### Mean environmental conditions

**Figure 1:**
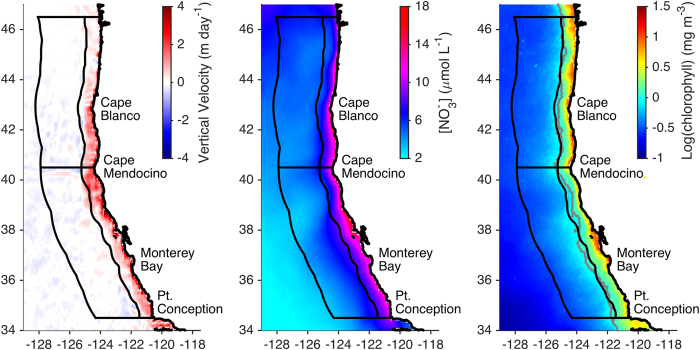
Study region. 1998–2010 March-August means of (left) model vertical velocity at the base of the mixed layer, (middle) nitrate concentration at the base of the mixed layer, estimated from model hydrography and observed temperature-salinity-nitrate relationships (Fig. S5), and (right) SeaWiFS surface chlorophyll concentration. Details of variable calculations are provided in the Methods. Black contours divide the CCS domain into northern (40.5–46.5°N) and central (34.5–40.5°N) as well as nearshore (0–75 km from shore) and offshore (75–300 km from shore) regions. The gray contour indicates surface chlorophyll concentration of 1 mg m^−3^. Figure created using MATLAB R2015a (www.mathworks.com).

The environmental setting during the central/northern CCS upwelling season, as estimated from data-assimilative model output and satellite observations, is shown in Fig. 1. A nearshore band extending ~75 km offshore is characterized by mean vertical velocities of several meters per day at the base of the mixed layer, nitrate concentrations of ~5–15 μmol L^−1^ at the base of the mixed layer ([NO_3_]_MLD_), and surface chlorophyll concentrations ([chl]) greater than 1 mg m^−3^. Surface chlorophyll and [NO_3_]_MLD_ in the offshore region (75–300 km from shore) are significantly lower than in the nearshore region but are still higher than concentrations in the oligotrophic subtropical gyre. Vertical velocities in the offshore region are weak and of variable sign, and much of the offshore nutrient and phytoplankton biomass is derived through advection from the nearshore region rather than from local processes^16^.

In the alongshore direction, a marked change in the coastal orientation at Cape Mendocino (~40.5°N) divides the domain into central and northern CCS regions, which experience distinct patterns of atmospheric forcing^21^. The central CCS has generally stronger upwelling and higher [NO_3_]_MLD_ than the northern CCS, however [chl] is higher in the northern region (Fig. 1). This discrepancy is especially pronounced north of Cape Blanco and is likely due to regional geographic features including the Strait of Juan de Fuca, the Columbia River, and a relatively wide shelf, all of which facilitate nutrient delivery to the coastal zone^22^.

### Environmental control of phytoplankton biomass

**Figure 2:**
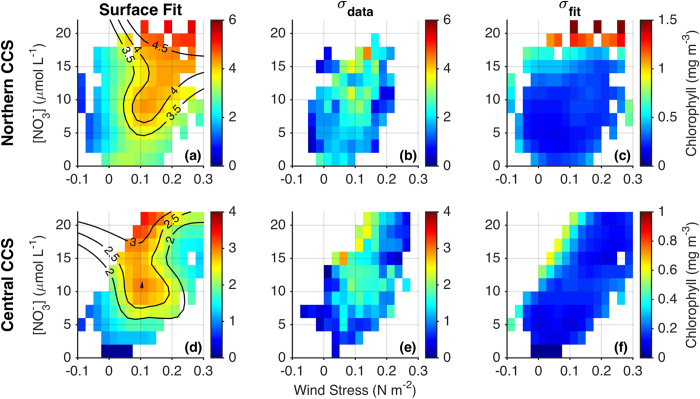
Chlorophyll dependence on wind stress and nitrate in the nearshore region. (**a,d**) Surface chlorophyll concentration, averaged from the coast to 75 km offshore, is shown as a function of alongshore wind stress (equatorward is positive) and nitrate concentration at the base of the mixed layer in the northern and central CCS regions. Alongshore wind stress is measured 75 km offshore and nitrate concentration at the base of the mixed layer is averaged over the 75 km coastal band (see Fig. 1). All variables are 8-day averages with a subsequent three-point moving average applied, increasing the effective temporal averaging to 24 days. (**b,e**) Standard deviations of data points within each pixel indicate spread in the data. (**c,f**) Standard deviation of 1000 surface fits, each performed with 50% of the data randomly withheld, indicates uncertainty in the surface fits. For (**c**,**e**), white pixels have fewer than 3 data points; for other panels white pixels indicate no data. Note smaller [chl] ranges in rightmost panels.

**Figure 3:**
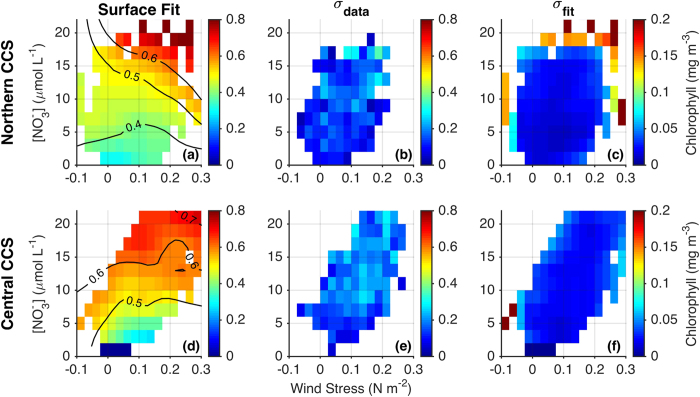
Chlorophyll dependence on wind stress and nitrate in the offshore region. As in Fig. 2, but for chlorophyll averaged over the offshore region (75–300 km from shore).

The dependence of [chl] on alongshore wind stress (τ^a^) and [NO_3_]_MLD_ is shown in Fig. 2 for the nearshore region (0–75 km from shore) and in Fig. 3 for the offshore region (75–300 km). Approximately 1,000 data points, each representing a spatial average over the nearshore or offshore region (Figs S1 and S2), were used to construct each of the fits shown in Figs 2 and 3. Data were limited to the upwelling season (March–July for the central CCS, April–August for the northern CCS), and each data point is an 8-day mean with an additional 3-point moving average applied, for an effective temporal averaging of 24 days (see Methods for additional details). While the 3-point moving average eliminates some spurious results (e.g., at high wind stress in the nearshore Central CCS), it does not qualitatively change our findings (Fig. S3), suggesting that these relationships hold for time scales of ~1 week–1 month. Nonetheless, it is important to consider the spatiotemporal averaging of our data when interpreting results, as it may hide important details on shorter time scales (e.g., brief relaxation of the wind) and finer spatial scales (e.g., retention areas, headlands). Note that while satellites only observe chlorophyll near the ocean surface, near-surface chlorophyll is highly correlated with depth-integrated chlorophyll off the California coast (r^2^ = 0.9)^23^. We therefore use [chl] derived from satellite interchangeably with phytoplankton biomass throughout this paper. Also, it is important to note that we are not using [chl] as a proxy for primary productivity, which is just one contributor to the phytoplankton biomass relationships in Figs 2 and 3.

While phytoplankton biomass is generally higher in the northern CCS than in the central CCS, its relationship to wind stress and nitrate availability is remarkably similar between the two regions. In the nearshore region, the optimal wind stress for maximal [chl] is ~0.1 N m^−2^ in the central CCS and ~0.1–0.2 N m^−2^ (depending on background nitrate concentration) in the northern CCS (Fig. 2a,d). Chlorophyll is limited when wind stress is weaker or stronger than the optimal value, presumably due to nutrient limitation and consequent reduced productivity at low wind stress and physical processes (offshore advection, subduction, mixed layer deepening) at high wind stress. The optimal wind stress of 0.1 N m^−2^ in the central CCS is equivalent to a wind speed of ~8.5 m s^−1^ (ref. 24), falling between optimal wind estimates of ~11.5 m s^−1^ for shelf productivity in a simple model^17^, and 5–6 m s^−1^ for pelagic fish recruitment^25^. However, Fig. 2 also shows that identification of an optimal wind intensity tells an incomplete story relative to [chl]. Subsurface nitrate availability can also exert strong control over the biomass attainable at a given wind stress. In the presence of optimal wind stress, [chl] dependence on nitrate is especially strong below [NO_3_]_MLD_ ≈ 10 μmol L^−1^. In the central CCS for example, when τ^a^ = 0.1 N m^−2^, an increase in [NO_3_]_MLD_ from 5 to 10 μmol L^−1^ results in ~50% higher [chl] (Fig. 2d). In other cases (e.g., τ^a^ ≈ 0 and τ^a^ ≈ 0.2 N m^−2^ in the central CCS), [NO_3_]_MLD_ appears to exert little influence over [chl], suggesting that if upwelling is too weak or lateral advection and/or subduction too strong, [chl] is similarly limited regardless of nitrate availability. Finally, there exists a small window of wind stress for which the relationship between [NO_3_]_MLD_ and [chl] is non-monotonic. For example, at τ^a^ = 0.1 N m^−2^ in the northern CCS, [chl] increases up to [NO_3_]_MLD_ ≈ 10 μmol L^−1^, decreases from [NO_3_]_MLD_ ≈ 10 to 15 μmol L^−1^, and then increases again for [NO_3_]_MLD_ > 15 μmol L^−1^. We suspect this special case is an artifact as we cannot speculate on what mechanisms would produce such a pattern.

In the offshore region, [chl] is much less sensitive to alongshore wind stress than it is in the nearshore region (Fig. 3a,d). A weak positive relationship between wind stress and [chl] suggests lateral export of nutrients and/or phytoplankton from the nearshore zone during strong wind events, however reductions in nearshore biomass are not compensated by increases offshore. This finding is consistent with an overall limitation of surface mixed layer productivity in high winds, potentially due to light limitation in a deep mixed layer^8^ or to subduction of nutrients and phytoplankton^13^. There is however a much stronger correlation between offshore [chl] and [NO_3_]_MLD_. This relationship may be causative; i.e., elevated offshore [chl] is supported by lateral advection and subsequent uptake of nitrate upwelled near the coast^16^. Similarly, high nitrate in this case may serve as a proxy for iron upwelled from the continental shelf, which can have a critical role in regulating offshore productivity^26^. Alternatively, the correlation between nearshore [NO_3_]_MLD_ and offshore [chl] may indicate a common driver for both, where conditions that produce elevated nitrate nearshore (e.g., deep mixing) do the same offshore. Offshore [chl] may also be moderated by wind stress curl driven productivity^27^, though there is no significant correlation between [chl] and the magnitude of wind stress curl in the offshore region (r = −0.02 and −0.05 in the northern and central CCS, respectively).

For each [chl] surface fit to τ^a^ and [NO_3_]_MLD_ shown in Figs 2 and 3, we provide accompanying estimates of scatter in the data (σ_data_) and uncertainty in the fit (σ_fit_). The former is the standard deviation of [chl] data within each pixel of the τ^a^ -[NO_3_]_MLD_ parameter space (see Fig. S1 for scatter plots of all data points), while the latter is the standard deviation of 1,000 fits to the data, each performed with 50% of the data randomly withheld. Scatter in the data (σ_data_) is similar in magnitude to the fit itself, indicating substantial unexplained variability due to the many factors outside of wind stress and subsurface nitrate concentration that can influence phytoplankton biomass. Uncertainty in the fits themselves is much smaller, with σ_fit_ typically an order of magnitude smaller than σ_data_. The lowest values of σ_fit_ occur in data-rich areas of the parameter space, and the diagonal distribution of available data (Fig. S2) as well as σ_fit_ (especially in Fig. 2f) results from a positive correlation between τ^a^ and [NO_3_]_MLD_. Conversely, the largest uncertainties tend to occur in data limited areas of the parameter space, typically at extreme values of τ^a^ and [NO_3_]_MLD_ or where their decoupling is most pronounced. Correlation coefficients for the fits in Figs 2a,d, and 3a,d are 0.38, 0.39, 0.49, and 0.47, respectively. The substantial fraction of unexplained variance highlights the influence of other, unaccounted for, variables (detailed in Methods). Importantly, variables such as irradiance and day length have pronounced seasonal cycles and can drive changes in [chl] independent of wind strength or nutrient availability, even within the upwelling season. Chlorophyll predictions based on the fits in Figs 2 and 3 therefore underestimate the observed variance (Fig. S4), and we suggest that they are best used in two ways: (i) to quantify the wind/nitrate space most conducive to high chlorophyll concentrations, and (ii) to predict chlorophyll anomalies related specifically to forcing by wind and nitrate variability (in other words, to predict chlorophyll anomalies relative to the interannual variance; see ‘Chlorophyll predictions’ in the Methods). The latter is demonstrated in the following sections, in which the relationships of Figs 2 and 3 are used as a framework for interpreting past events when environmental conditions and phytoplankton responses departed significantly from the climatological state.

### Delayed upwelling in 2005

An unusually late shift to upwelling-favorable winds in 2005 had widespread impacts on the northern CCS ecosystem^28^, including anomalously warm sea surface temperatures^29^, low phytoplankton and zooplankton biomass^30^,^31^, low mussel and barnacle recruitment^4^, and dramatic changes in the populations and distributions of marine nekton^32^. Here we describe the environmental drivers of these effects using the wind stress-nitrate-chlorophyll relationships described by Figs 2 and 3.

**Figure 4:**
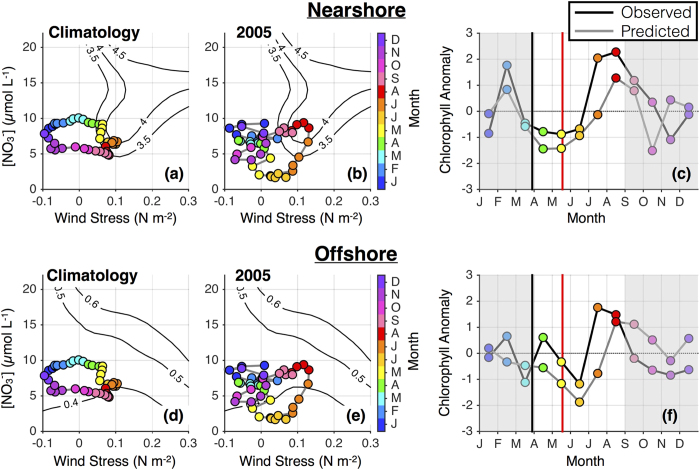
Delayed 2005 spring transition in the northern CCS. (**a,d**) Climatological and (**b,e**) 2005 annual progression of wind stress and nitrate concentration are shown for the (top) nearshore and (bottom) offshore regions of the northern CCS. Variables are calculated as in Fig. 2, and chlorophyll dependence on wind stress and nitrate for the nearshore (offshore) region is indicated by contours from Figs 2a and 3a. Chlorophyll anomalies are averaged over the (**c**) nearshore and (**f**) offshore regions and divided by the standard deviation of 1998–2010 monthly anomalies. Months outside of the upwelling season, which were not included when calculating the relationships in Figs 2 and 3, are shaded in gray. Black and red vertical lines mark the climatological and 2005 Spring Transition Indices, respectively, calculated from alongshore wind as described in Bograd *et al.*^65^.

Figure 4 shows the evolution of [NO_3_]_MLD_, τ^a^, and [chl] in the northern CCS in a climatological year (Fig. 4a,d) as compared to 2005 (Fig. 4b,e). In a climatological year, winds are poleward in the winter and turn equatorward (upwelling favorable) in March, with peak upwelling occurring in June. Spring upwelling draws deep nitrate-rich water toward the surface, counteracting the influence of solar heating that would otherwise tend to increase stratification, shoal the mixed layer, and inhibit nitrate availability. In 2005, however, alongshore winds remained weak and variable throughout the spring, while the mixed layer shoaled. As a result, [NO_3_]_MLD_ fell to concentrations near zero and phytoplankton biomass was anomalously low (Fig. 4c,f). Winds finally turned predominantly equatorward in mid May, marking a spring transition ~1.5 months later than normal. Initially, this shift in winds produced no significant response in [chl], as upwelled waters were nitrate poor. In July, a return of τ^a^ to near or above climatological values drove a rapid subsequent increase in [NO_3_]_MLD_ and stimulated a significant biological response evident in elevated [chl]. The wind-nitrate-chlorophyll relationship shown in Fig. 2a,d predicted the suppression of phytoplankton biomass in spring and early summer as well as a late summer shift to favorable conditions that produced anomalously high biomass (Fig. 4c,f), supporting the paradigm of bottom-up control by wind and nitrate availability.

Though the seasonal cycle of northern CCS upwelling was highly anomalous in 2005, cumulative wind stress over the entire year was similar to climatological values. The same can be said for mean annual [chl], indicating that while the spring transition was late and the biological response lagged by an additional month or more^33^ (Fig. 4), net impacts on phytoplankton biomass were minimal. Similarly, a late season rebound in mussel recruitment off Oregon led to normal overall recruitment in 2005 despite extremely poor recruitment in the early season^4^, and nekton generally rebounded by September 2005^32^. However, strong late season recruitment did not compensate for poor early season recruitment of barnacles^4^ and zooplankton biomass remained suppressed throughout 2005 and into 2006^31^. The response of higher trophic levels to anomalous environmental conditions is therefore highly varied across species, and in this case is likely influenced by phenological mismatches between predator and prey.

### Anomalous subarctic influence in 2002

The upwelling season of 2002 was characterized by unusually cold and fresh waters occupying the upper halocline (30–150 m) off the U.S. west coast, with temperature and salinity anomalies off Oregon approximately equal in magnitude and of opposite sign to those observed during the 1997–1998 El Niño^34^. The proximate cause of these anomalies was wind-driven change in the northeast Pacific circulation; in particular, enhanced southward advection of nutrient-rich subarctic water stimulated high primary productivity, especially in the northern CCS (ref. 35 and references therein). As in the previous section, we use the framework of Figs 2 and 3 to examine anomalous phytoplankton concentrations in the context of environmental drivers.

**Figure 5:**
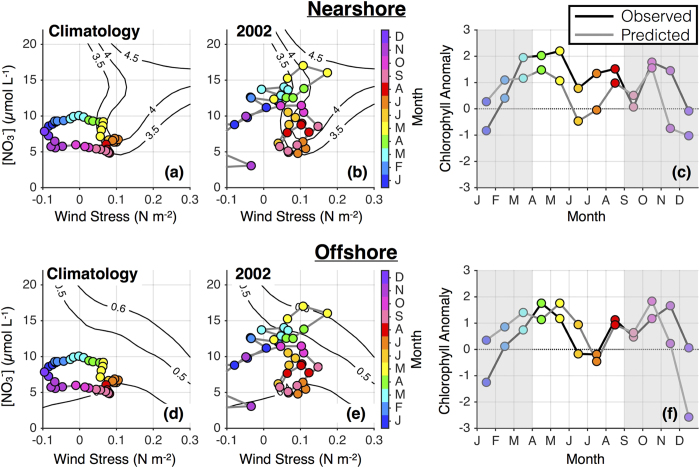
Anomalous influence of nutrient-rich subarctic waters in 2002. Line and contour plots are as in Fig. 4, but for 2002.

The progressions of τ^a^, [NO_3_]_MLD_, and [chl] anomalies during 2002 are shown in Fig. 5. Elevated nitrate concentrations arrived in January, reaching levels ~50% higher than normal in the spring, and persisted throughout much of the year. Upwelling favorable winds were also slightly stronger than normal in the spring and late summer, though wind anomalies were much less pronounced than those in the nitrate field. The combination of moderate winds and high nitrate proved ideal for phytoplankton, supporting very high spring and late summer biomass in both the nearshore and offshore environments. Our analysis accurately predicted observed patterns in chlorophyll variability (Fig. 5c,f), though underestimation of positive nearshore [chl] anomalies suggests that we overestimate the pernicious influence of low wind stress (<0.1 N m^−2^) in the presence of high nitrate concentrations (Fig. 5b,c).

The conditions of 2002 highlight the importance of understanding both local and remote influences when studying biological responses to the environment. In contrast to 2005, when anomalies in the nutrient field were tied to local winds, 2002 brought positive nitrate anomalies to the northern CCS through lateral advection of subarctic water. Because high nitrate concentrations were available immediately beneath the mixed layer, moderately strong local upwelling was able to efficiently supply the surface mixed layer with nutrients, stimulating considerable primary production. This modulation of phytoplankton biomass by advective processes underscores the strength of considering atmospheric forcing and source water properties together when assessing their impacts on the ecosystem.

### El Niño/La Niña events of 1998–1999

The 1997–1998 El Niño was by some metrics the strongest on record^36^, and was followed by a multi-year La Niña event that signaled a regime shift in the north Pacific climate^37^,^38^. Ecosystem impacts in the CCS from physics to top predators are well documented (see special issue of Progress in Oceanography, Volume 54, 2002). Again, we place the temporal evolution of the physical and biogeochemical environment in the context of bottom-up controls detailed in the present study.

**Figure 6:**
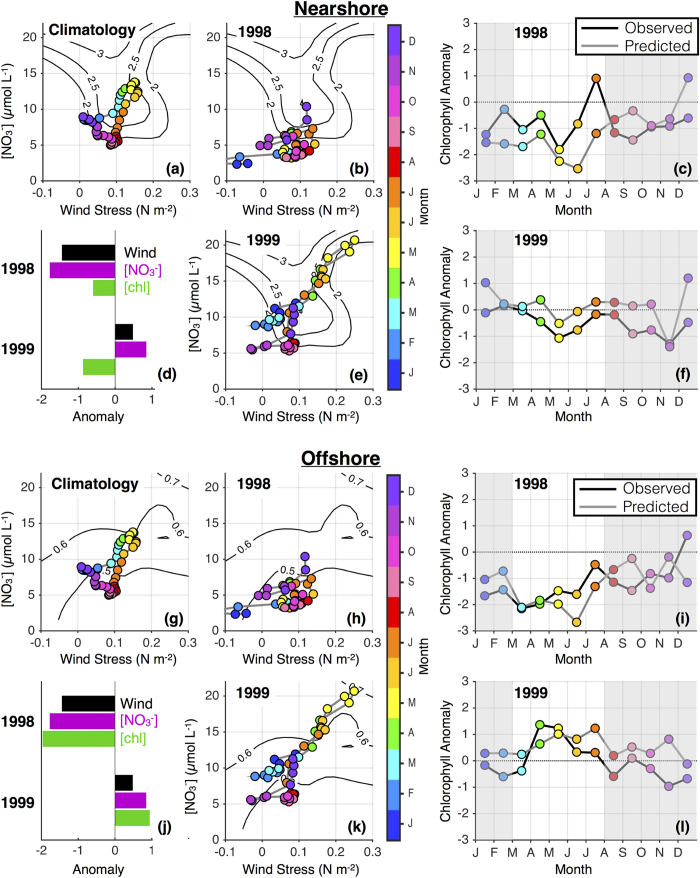
The 1998–1999 El Niño/La Niña cycle in the central CCS. Line and contour plots are as in Figs 4 and 5, but for (**a–f**) nearshore and (**g–l**) offshore regions of the central CCS in 1998–1999. Chlorophyll contours are from Fig. 2d for nearshore (**a,b,e**) and Fig. 3d for offshore (**g,h,k**) plots. Bar plots (**d,j**) summarize March-July mean anomalies for 1998 and 1999, normalized by the standard deviation of March-July means for 1998–2010.

Environmental conditions in 1998 and 1999 and their relations to nearshore and offshore chlorophyll concentrations are shown in Fig. 6. El Niño was near peak strength in January 1998, and communication of tropical anomalies through oceanic propagation and atmospheric teleconnection drove strong poleward winds and extremely low [NO_3_]_MLD_ in the CCS^39^. Spring and early summer winds were also weaker than normal (Fig. 6a,b), but actually of optimal magnitude to produce high [chl] in the nearshore region given adequate nitrate in the subsurface. However, remote forcing by equatorial and coastal wave propagation from the tropics produced an exceptionally deep nitracline and therefore low [NO_3_] in upwelling source waters. This effect was exacerbated by anomalously weak local winds and a relatively shallow source depth for upwelling^39^, resulting in upwelling season [NO_3_]_MLD_ values of ~3–7 μmol L^−1^, well below climatological values of ~10–15 μmol L^−1^ (Fig. 6a,b). Observed phytoplankton biomass was suppressed in both the nearshore and offshore regions, as predicted based on the influences of wind and subsurface nitrate (Fig. 6c,i).

The switch from El Niño conditions in 1997–1998 to La Niña conditions in 1998–1999 is typically regarded as a return to high productivity. However, while [chl] was uniformly low in 1998, anomalies in 1999 were spatially varied in the cross-shore direction. Early in 1999, [NO_3_]_MLD_ was much higher than at the same time in 1998, again consistent with nitracline depth anomalies driven by anomalous atmospheric and basin-scale oceanic forcing during El Niño and La Niña. Similarly, alongshore winds strengthened considerably in 1999, especially in May when τ^a^ reached levels ~60% higher than climatological values (Fig. 6a,e). Thus, the combination of remote and local influences produced a shallow nitracline and a deep source for upwelling, and [NO_3_]_MLD_ climbed as high as 20 μmol L^−1^ in May (~50% higher than the climatological concentration), providing ample nitrate supply to the surface mixed layer. However, such strong wind also drove rapid offshore advection and intense mixing, and Fig. 6e,f suggests that despite elevated nitrate levels, nearshore [chl] in spring/summer was limited by excessive wind stress. Conversely, the conditions of 1999 were optimal for the development of high [chl] offshore, which benefitted from high nitrate concentrations (Fig. 6k,l). Observations from the central CCS in the spring/summer of 1999 support this paradigm; new production anomalies were negative nearshore and positive offshore^40^, elevated chlorophyll extended unusually far offshore^41^, and reductions or offshore displacements of zooplankton and juvenile fish were attributed to rapid offshore advection driven by strong upwelling^42^,^43^. Predictions based solely on wind and subsurface nitrate capture the anomalously high [chl] offshore (Fig. 6l) and lower anomalies nearshore, though the adverse effects of high winds appear to be underpredicted for April-June (Fig. 6f).

Interestingly, nearshore [chl] was similarly limited in 1998 and 1999, though by completely different mechanisms (Fig. 6d). In 1998, τ^a^ was anomalously weak, there was a deep nitracline associated with remote forcing from the tropical El Niño, and resultant low [NO_3_]_MLD_ values are implicated in [chl] limitation. In 1999, [NO_3_]_MLD_ was exceptionally high but nearshore [chl] in the spring and early summer was limited by strong winds. Substantial differences in overall system biomass between the two years were therefore driven almost entirely by anomalies of opposite sign in the offshore environment (Fig. 6j), which may be influenced by local curl-driven upwelling or by offshore advection of nearshore nutrients and phytoplankton.

## Discussion

In this paper, we present a framework for evaluating bottom-up influences on ecosystem functioning in an Eastern Boundary Upwelling System. We find moderate wind stress to be optimal for accumulation of phytoplankton biomass in the nearshore environment and in the CCS as a whole. Productivity is nutrient limited below the optimal wind stress, while at higher wind stress physical processes (offshore advection, subduction, enhanced mixing) conspire to export nutrients and organic matter either offshore or below the euphotic zone. Conversely, the offshore region appears relatively unaffected by both nearshore wind stress and offshore wind stress curl. In both the nearshore and offshore environments, [chl] correlates positively with subsurface nitrate concentration. These patterns are robust across the dynamically different central and northern CCS regions and constitute our primary result: the isolation of fundamental relationships between wind, subsurface nitrate, and chlorophyll that emerge amidst many confounding influences (see Methods).

While phytoplankton biomass exhibits relationships with both physical (wind stress) and chemical (nitrate) forcings individually, a key result of our study is that the two have strong interactions in terms of their influences over [chl]. First, nitrate concentration at the base of the mixed layer is dependent on the wind history and its modification of the water column, as well as remotely forced changes in the subsurface nitrate field. The utility of instantaneous wind stress alone as an indicator of potential productivity is therefore limited, even though stronger winds generally correlate to higher nitrate concentrations. Explicit representation of subsurface nitrate in our study negates the need for proxies such as cumulative wind stress and implicitly accounts for changes deeper in the water column structure associated with basin scale climate variability and lateral advection. We are therefore able to explain anomalous events that are driven largely by remote forcing (e.g., the deep nitracline in 1998, anomalous equatorward advection of subarctic waters in 2002) or by local forcing (e.g., weak/delayed winds in 2005). Second, a lag on the order of a week to a month often exists between a change in alongshore winds (e.g., the start of the upwelling season) and a measurable biological response. Our results suggest that this lag lies primarily in the response of the nutrient field to wind forcing (e.g., Fig. 4b), and that the phytoplankton response is fast (<1 week) once both the wind and nutrient conditions are right. Third, in terms of altering surface chlorophyll concentrations, the impact of changes in either winds or nitrate is dependent on the state of the other. For example, reducing wind stress in the central CCS from 0.2 to 0.1 N m^−2^ would on average produce a ~50% increase in nearshore [chl] when [NO_3_]_MLD_ ≈ 15 μmol L^−1^, but the same reduction in wind stress would produce no discernible change in [chl] when [NO_3_]_MLD_ ≈ 5 μmol L^−1^ (Fig. 2d). Fourth, a single metric that combines subsurface nitrate and vertical transport (i.e., vertical nitrate flux) is inadequate for characterizing bottom-up control of phytoplankton. For example, weak upwelling of nitrate-rich water and strong upwelling of nitrate-poor water may produce the same vertical nitrate flux. However, the biological response is very different, with the latter characterized by a deep mixed layer, rapid offshore advection, and suppressed phytoplankton biomass. For all of these reasons, understanding the state of both the winds and the subsurface nitrate field is critical to understanding bottom-up impacts on phytoplankton. The examples of 2005, 2002, and 1998–1999 are cases where the biological response to environmental conditions cannot be interpreted based on either wind or nutrient data alone. Similar effects on phytoplankton biomass can result from several distinct mechanisms; for example, we have shown cases where anomalously low nearshore [chl] was driven by a deep nitracline (1998), unusually strong winds (1999), and an interaction whereby anomalously weak equatorward winds caused a drop in subsurface nitrate (2005).

Finally, our results can be used to contextualize potential ecosystem responses to future changes in the CCS. Bakun^44^ proposed a mechanism for increased upwelling-favorable winds in a warming world and while the existence of such a trend and its governing dynamics have fueled much debate in recent years, the most recent retrospective analyses and model forecasts suggest that the sign and magnitude of long-term trends in upwelling winds are likely latitude and region dependent^45^,^46^,^47^. Our results suggest that intensification of peak upwelling season winds would make them stronger than the optimal value for primary producers in the nearshore environment (Figs 4a and 5a). However, such an increase should also enhance nutrient delivery to the surface mixed layer^20^, increasing biomass in the offshore region and at least partially offsetting the negative impact of strong winds on the nearshore region. A wind intensification scenario would also produce optimal wind stress earlier in the year, resulting in an earlier onset and longer duration of the high productivity season. In the case of weakened alongshore winds, summertime productivity in the central CCS could actually be enhanced, provided subsurface nitrate remains near climatological concentrations (Fig. 5a). Alternatively, a dramatic increase in the nitrate concentration of upwelling source waters (e.g., a doubling by 2100)^48^ would likely negate any impact of changes in the winds and result in a highly productive environment (Figs 2 and 3). Ultimately, ecosystem impacts arising from each of these scenarios will differ greatly as individual species suffer or prosper based on their sensitivities to the overall abundance of phytoplankton, its phenology, and its spatial distribution.

## Methods

### Study Domain

Our study domain spans the west coast of the US from Point Conception in the south to southern Washington State in the north (34.5–46.5°N). As in previous studies (e.g., ref. 21), we split this region at Cape Mendocino (40.5°N) into central CCS and northern CCS domains. In the cross-shore direction we define a nearshore region (0–75 km from shore) characterized in the upwelling season by strong vertical velocities and surface chlorophyll concentrations greater than 1 mg m^−3^ (Fig. 1), and an offshore region (75–300 km from shore) roughly corresponding to the California Current transition zone^26^. Our analysis spans the years 1998–2010, the period of SeaWiFS data availability. As the focus of the study is chlorophyll dependence on wind stress and nitrate concentration, we focus on the upwelling season (March-July for the central CCS, April-August for the northern CCS), when physical transport and nutrient supply are expected to be dominant regulators of phytoplankton biomass. Outside of the upwelling season other processes are likely more important; light limitation in winter months may limit chlorophyll even in the presence of optimal wind and nutrient conditions^14^, while in the fall the phytoplankton assemblage is dominated by picoplankton^49^ and vertically migrating dinoflagellates^50^, which thrive in warm, stratified conditions and are not dependent on upwelling.

### Chlorophyll Data

Satellite chlorophyll estimates are from the Sea-viewing Wide Field-of-view Sensor (SeaWiFS) with the NASA/GSFC OC4v4 algorithm^51^. Global daily composite fields, with spatial resolution of 1/12°, were downloaded from NOAA CoastWatch.

### Ocean Model

Wind stress, temperature, salinity, and mixed layer depth were obtained from a historical analysis of the CCS that uses the Regional Ocean Modeling System (ROMS) with 4-Dimensional variational (4D-Var) data assimilation. The analysis spans 1980–2010 and is described in detail elsewhere^52^,^53^. Surface radiative and freshwater fluxes were derived from the European Centre for Medium-Range Weather Forecasting (ECMWF) 40-year reanalysis (ERA-40)^54^ prior to 2002 and from ERA-Interim^55^ for 2002–2010. Lateral boundary conditions were taken from the Simple Ocean Data Assimilation (SODA) reanalysis^56^. For the period of this study (1998–2010), wind forcing was derived from the Cross Calibrated Multi Platform (CCMP) product^57^. Data assimilation was performed in 8-day cycles. In each cycle the initial conditions, boundary conditions, and surface forcing were adjusted by the 4D-Var system to improve model representation of observed dynamics^58^,^59^,^60^. Assimilated data include available satellite Sea Surface Temperature (AVHRR, AMSR-E, and MODIS Terra) and Sea Surface Height (AVISO) as well as *in situ* salinity and temperature measurements from the ENSEMBLES (EN3) database.

### Nitrate Model

Nitrate concentration at the base of the mixed layer was calculated as follows: First, available data from the World Ocean Database and the Global Ocean Ecosystem Dynamics (GLOBEC) were used to fit nitrate as a function of temperature and salinity using the MATLAB function gridfit (http://www.mathworks.com/matlabcentral/fileexchange/8998) with a smoothness parameter of 1.5 and 20 nodes in the x and y directions. Temperature-salinity-nitrate fits were constructed separately for the central and northern regions using only data from the upwelling season in order to minimize latitudinal and seasonal biases^61^. Data were further limited to the upper 200 m of the water column and the years of our study (1998–2010). In all, 1049 measurements in the central CCS and 3772 measurements in the northern CCS were used to construct the nitrate relationships, which capture 97% and 91% of the observed variance, respectively (Fig. S5). Next, mixed layer depth in the model was estimated from the temperature and salinity fields according to Kara *et al.*^62^. Model temperature and salinity at the base of the mixed layer, along with the nitrate fits in Fig. S5, were then used to estimate nitrate concentration at the base of the mixed layer. Validation of our model-based nitrate estimates, using independent training and validation datasets, is shown in Fig. S6. The model-based estimates capture 76% of the observed variance in subsurface nitrate concentration. Note that this validation accounts for uncertainty in both the model representation of subsurface physical properties and the relationship of those properties to subsurface nitrate concentration.

### Determining chlorophyll dependence on wind stress and nitrate

Surface chlorophyll concentration ([chl]) was fit as a function of alongshore wind stress (τ^a^) and nitrate concentration at the base of the mixed layer ([NO_3_]_MLD_. This process is outlined below for one example region (offshore in the northern CCS, i.e., Fig. 3a). These steps were repeated to define the wind stress-nitrate-chlorophyll relationship for each of the four regions in Figs 2 and 3 (nearshore/offshore and northern/central CCS).

1. Each variable (τ^a^, [NO_3_]_MLD_, and [chl]) was averaged over 8-day cycles coincident with the assimilation cycles of the ROMS reanalysis. The 8-day averaging period is consistent with typical timescales for upwelling events (~3–10 days^63^), and for phytoplankton response to an injection of upwelled nutrients (~3–7 days^64^). A three-point moving average was then applied to the 8-day averages, increasing the effective temporal averaging to 24 days.
2. The region of interest (for example, offshore in the northern CCS) was further divided into 1° latitude bins. Within each bin, τ^a^ was calculated 75 km from shore and [NO_3_]_MLD_ was averaged from the coast to 75 km from shore, in order to capture the coastal upwelling influence (Fig. 1). Chlorophyll was averaged over the cross-shore domain of interest (for example, 75–300 km from shore) only if spatial coverage of chlorophyll data was greater than 90%. When chlorophyll coverage is lower, spatial averages become less reliable, particularly with respect to biases in the nearshore region (Fig. S7). We used averages in 1° bins instead of the full 6° region to maximize the number of points with adequate chlorophyll coverage. Variability among 1° bins also allows for more complete coverage of the parameter space when fitting [chl] to τ^a^ and [NO_3_]_MLD_. Note that while [chl] was averaged over two different cross-shore regions, τ^a^ was always calculated 75 km from shore and [NO_3_]_MLD_ was always averaged from the coast to 75 km offshore. This approach allows us to determine the distinct nearshore and offshore chlorophyll responses to nitrate supplied by coastal upwelling (e.g., intense coastal upwelling may generate rapid offshore advection of upwelled nutrients and therefore high chlorophyll offshore but not nearshore).
3. Using data points generated from steps 1 and 2 (shown as scatter plots in Fig. S1), we fit a [chl] surface to τ^a^ and [NO_3_]_MLD_ using the MATLAB function gridfit (http://www.mathworks.com/matlabcentral/fileexchange/8998) with a smoothness parameter of 1.5 and 20 nodes in the x and y directions.

### Uncertainty estimates

We include with each surface fit in Figs 2 and 3 an estimate of variability in the data around the fit, as well as uncertainty in the fit itself. The former is calculated simply as the standard deviation of data points within each pixel, and is labeled as σ_data_. The latter is estimated with a bootstrap approach in which we fit the data 1000 times, each with 50% of the data randomly withheld. The standard deviation of the 1000 fits provides an estimate of uncertainty in the fit, and is labeled σ_fit_.

### Caveats

As detailed above, the [chl] fits to τ^a^ and [NO_3_]_MLD_, which form the basis of our analysis, capture only a moderate portion of the variance in [chl] (r = 0.38–0.49). There are many potential contributors to the unexplained variance, including influences of nutrients other than nitrate (e.g., iron, ammonium), zooplankton grazing, variable light levels and day length within the upwelling season, temporal and spatial autocorrelation of [chl], riverine influences on chlorophyll or on satellite estimates of chlorophyll, decoupling of surface and depth integrated chlorophyll, and uncertainty in our estimates of wind, nitrate, and surface chlorophyll. Given all of these confounding factors, the strength of our analysis is that we are able to extract robust fundamental relationships between wind, nitrate, and chlorophyll. The remarkable qualitative similarity of these relationships between the central and northern CCS speaks to their robustness, as many of the confounding processes listed above vary widely between the two regions. Nonetheless, care should be taken when extrapolating these relationships outside of the study period (1998–2010) as new observations may occur outside of the parameter space or violate assumptions of stationarity.

### Chlorophyll predictions

Chlorophyll predictions (Figs 4, 5, 6) were made by interpolating modeled τ^a^ and [NO_3_]_MLD_ values onto the fits in Figs 2a,d and 3a,d. These predictions were made on the same spatiotemporal scales as were used for the fits (i.e., 8-day means with additional 3-point smoothing, 1° latitude bins), and were subsequently averaged over the appropriate temporal (monthly) and spatial (e.g., northern CCS, nearshore) scales. Finally, chlorophyll anomalies for each month were normalized by the standard deviation of predicted chlorophyll across all years. Observed chlorophyll values were similarly averaged monthly and over the desired spatial domain and normalized by the standard deviation of observed chlorophyll values across all years.

## Additional Information

**How to cite this article**: Jacox, M. G. *et al.* Optimal Environmental Conditions and Anomalous Ecosystem Responses: Constraining Bottom-up Controls of Phytoplankton Biomass in the California Current System. *Sci. Rep.* **6**, 27612; doi: 10.1038/srep27612 (2016).

## Supplementary Material

Supplementary Information
