# Supplementary material for: Optimal Environmental Conditions and Anomalous Ecosystem Responses: Constraining Bottom-up Controls of Phytoplankton Biomass in the California Current System
Source: Sci Rep. 2016 Jun 9;6:27612. doi: 10.1038/srep27612 (PMC4899772; doi:10.1038/srep27612)
Supplement: Supplementary Information [file srep27612-s1.pdf]

Supplementary Information for:

Optimal Environmental Conditions and Anomalous Ecosystem Responses: Constraining Bottom-up Controls of Phytoplankton Biomass in the California Current System

Michael G. Jacox*[1,2], Elliott L. Hazen[2], and Steven J. Bograd[2]

[1]Institute of Marine Sciences, University of California, Santa Cruz, CA, USA

[2]Environmental Research Division, Southwest Fisheries Science Center, NOAA, Monterey, CA, USA

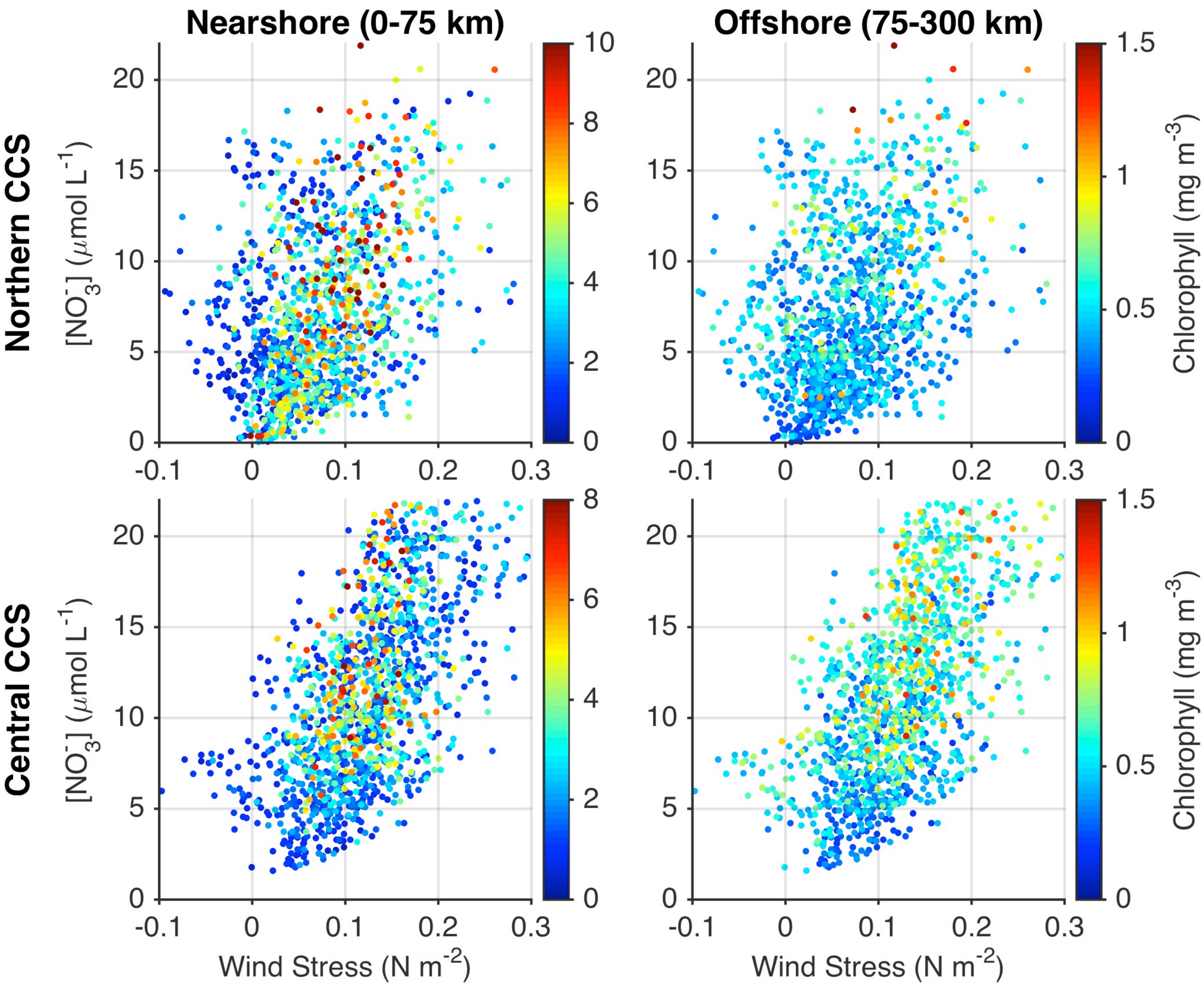

Figure S1: Environmental data. Scatter plots of all data points used in the surface fits of Figs. 2 and 3, one for each region (nearshore/offshore regions of the northern/central CCS). Each data point represents concurrent alongshore wind stress, nitrate concentration at the base of the mixed layer, and surface chlorophyll concentration, produced from 8-day means with a subsequent 3-point moving average (see Methods).

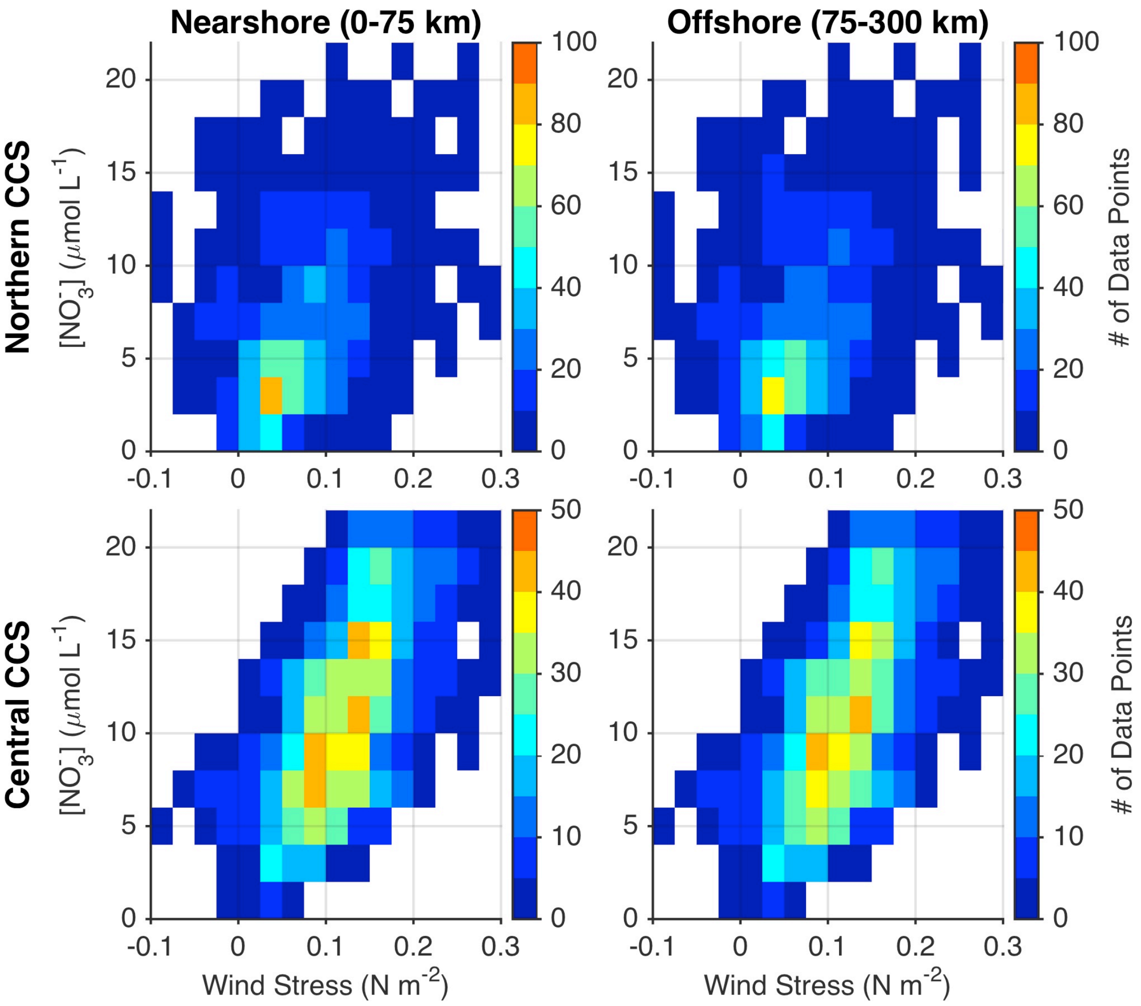

Figure S2: Distribution of Environmental data. For each region, color indicates the number of data points (from Fig. S1) that fall within each grid cell in the parameter space used for fits in Figs. 2 and 3. Note that the nearshore and offshore plots are nearly identical as they use the same wind stress and nitrate data and differ only in the number of data points that are excluded due to insufficient chlorophyll coverage in each of respective regions.

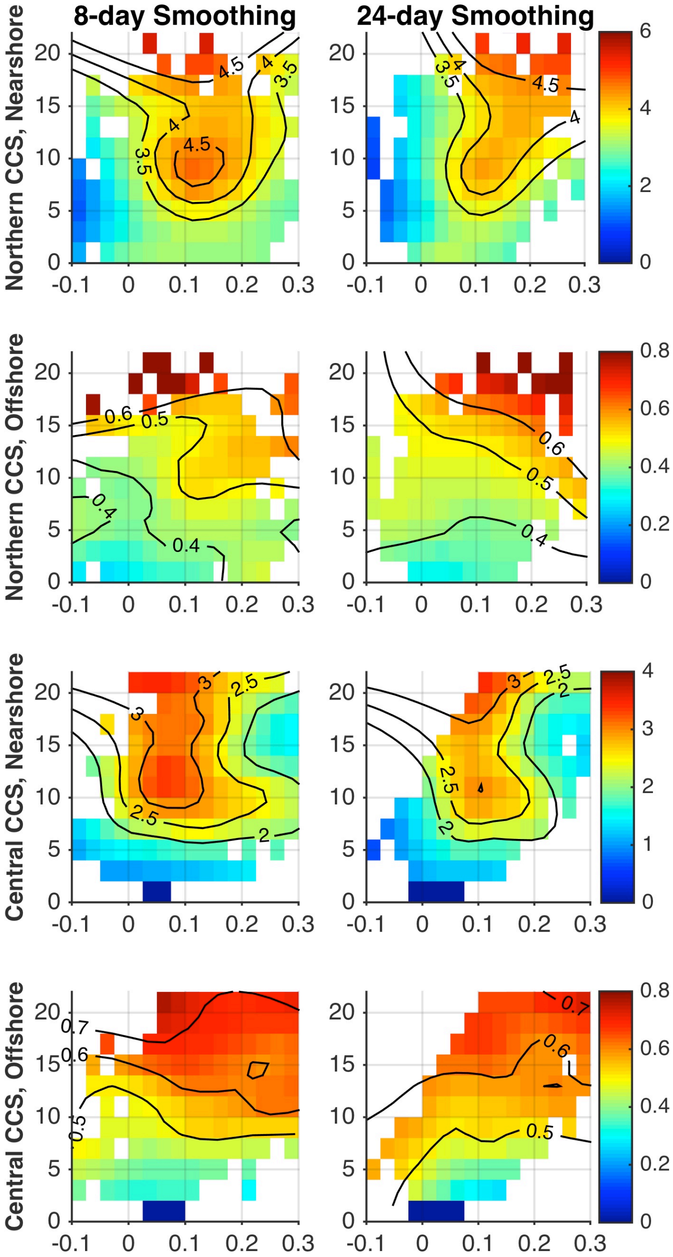

Figure S3: Effect of temporal smoothing on wind-nitrate-chlorophyll fits. Chlorophyll fit as a function of wind stress (N m^{-2}, x-axis) and nitrate concentration at the base of the mixed layer (μmol L^{-1}, y-axis) is shown before and after applying a 3-point moving average to the 8-day average data. Note that panels in the right column correspond with, from top to bottom, Fig. 2a, 3a, 2d, and 3d. Smoothing was applied to reduce unrealistic structure in regions of limited data availability (e.g., the feature at high wind stress and ~10 μmol L^{-1} for the nearshore central CCS).

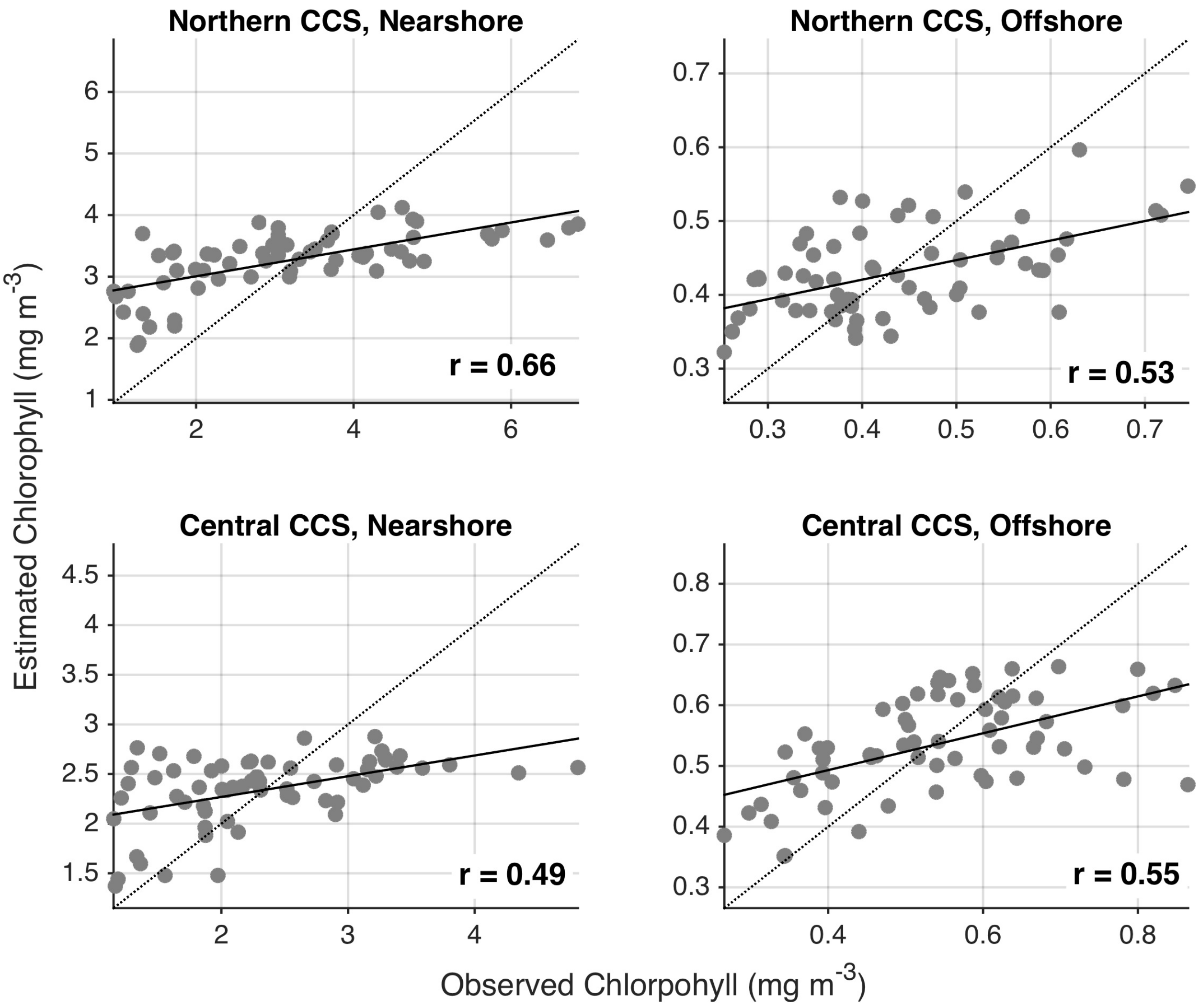

Figure S4: Monthly chlorophyll predictions vs. observations. Monthly chlorophyll predictions based on model wind and nitrate estimates and the framework of Figs. 2 and 3. Only months from the upwelling season are included. Note that chlorophyll predictions considerably underestimate the observed variance.

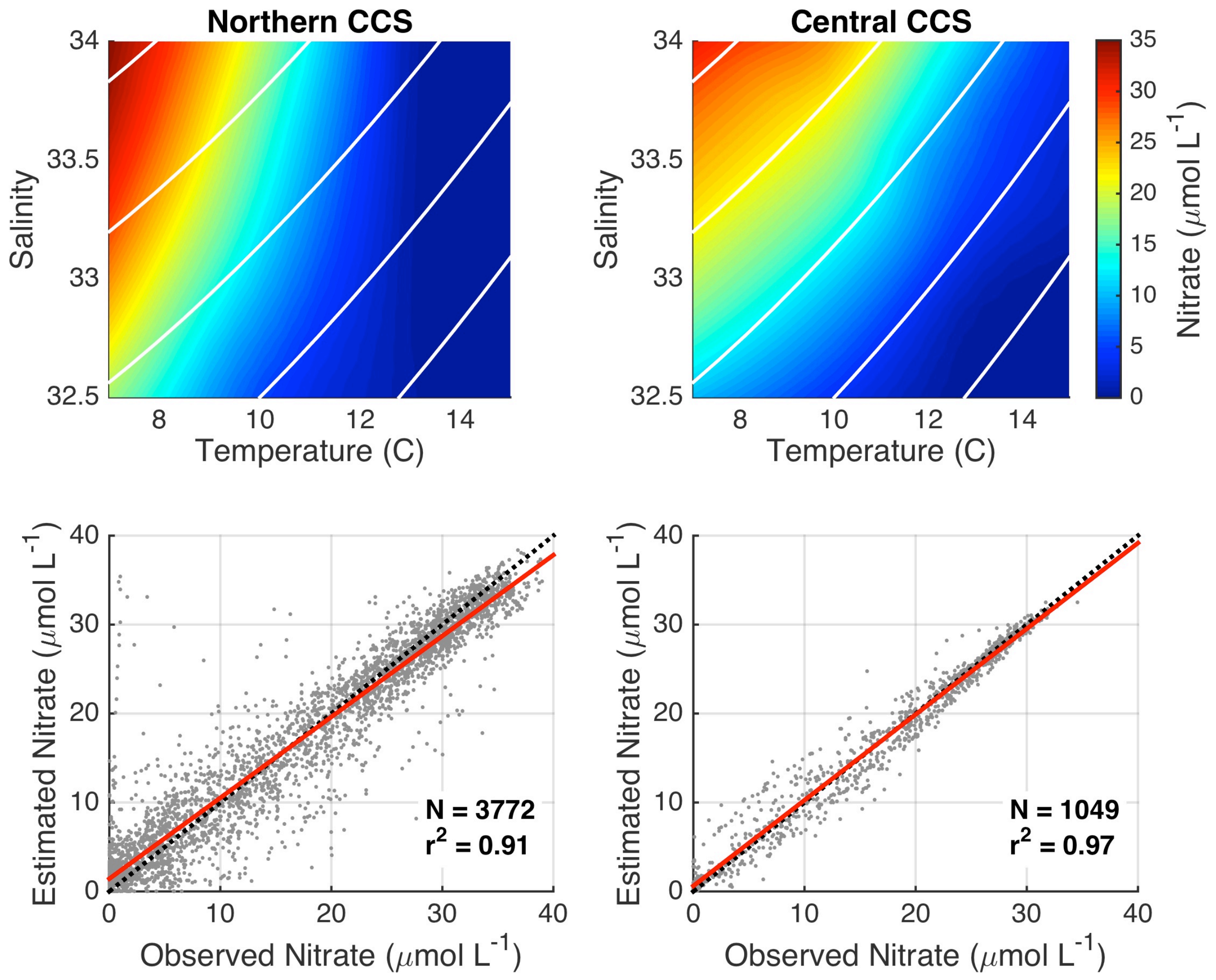

Figure S5: Nitrate model. (top) Temperature-salinity-nitrate relationships and (bottom) modeled vs. observed nitrate concentrations are shown for the northern and central CCS regions. Gray dots represent individual measurements, red lines are linear fits, and perfect agreement is marked by the dotted black 1:1 line.

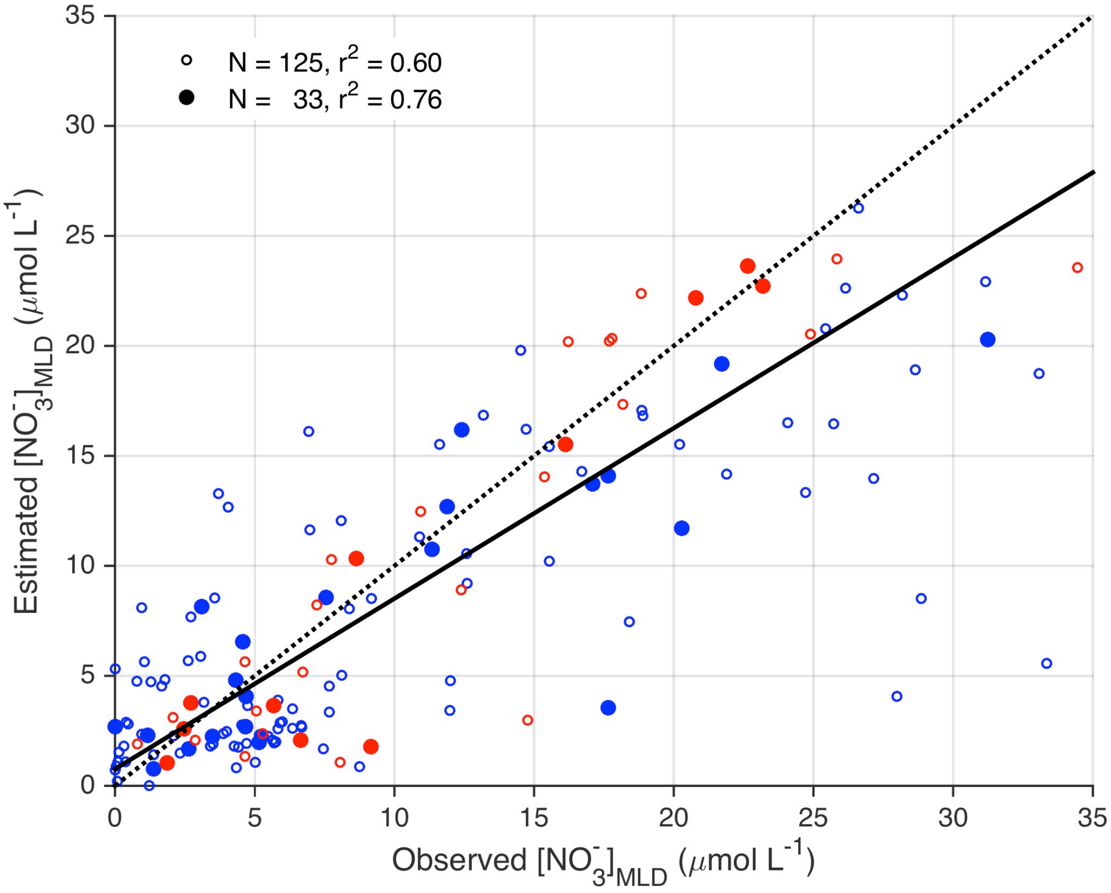

Figure S6: Validation of nitrate estimates. Available observational data was divided into northern (blue) and central (red) CCS regions and split 60/40 into training and validation datasets. Training data was used to produce temperature-salinity-nitrate fits as in Fig. S4. Validation data was then used to independently estimate nitrate at the base of the mixed layer from observations and from the model (using the nearest grid cell and time step). Open circles represent individual casts, while filled circles are averages of casts taken on the same day in close proximity (e.g., multiple nearshore stations of a CalCOFI line). The latter are more relevant for the spatially and temporally averaged data used in our anlaysis. The dotted black line is 1:1 while the solid line is a linear fit to the data.

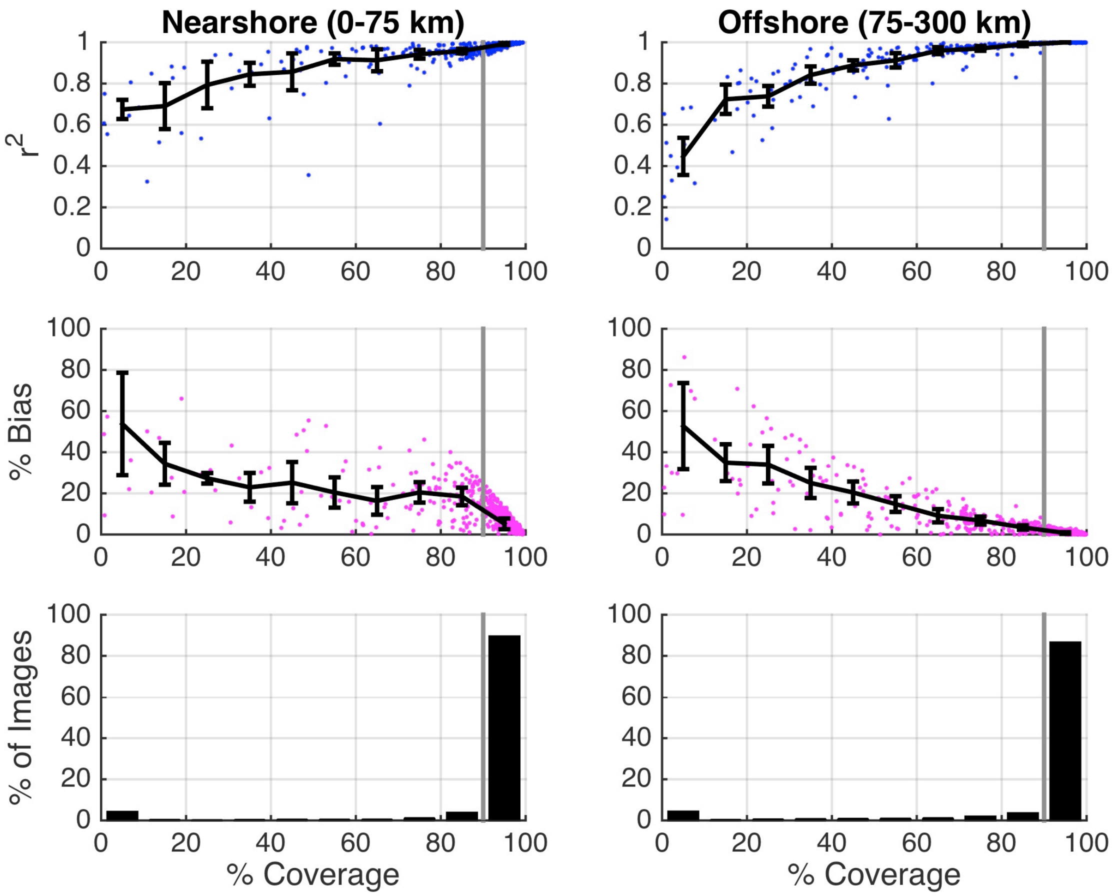

Figure S7: Impact of gaps in chlorophyll data on regional averaging. For each 1˚ latitude bin from 34.5˚N to 46.5˚N, we extract all upwelling season images (8-day means with subsequent 3-point moving average) with no data gaps and create a "cloud-free" time series. For the images with data gaps, we use each one to create a distinct cloud mask, and apply that mask to the cloud-free time series to create an "artificially cloudy" time series. Regional chlorophyll averages are then computed from the "cloud-free" and "artificially cloudy" time series, and their comparison yields one data point in the r^2 and bias plots. The number of data points in each panel is therefore the total number of chlorophyll images with incomplete spatial data coverage. Black lines and vertical bars indicate means and standard deviations, respectively, in 10% bins of coverage. Histograms at bottom show the distribution of data coverage in chlorophyll fields. The vertical gray line marks the 90% cutoff below which data are excluded from the analysis.
